# Impact of Osteoporosis Therapy in Geriatric Proximal Femur Fractures: A Retrospective Analysis from the German Registry for Geriatric Trauma (ATR-DGU^®^)

**DOI:** 10.3390/jcm15176916

**Published:** 2026-09-07

**Authors:** Henrik Teuber, Lisa Rockenbauer, Lara Zankena, Hannah Schmidt, Christoph Erichsen, Kai Oliver Jensen, Hans-Christoph Pape, Christian Hierholzer

**Affiliations:** 1 Department of Trauma Surgery, University Hospital Zurich, 8091 Zurich, Switzerland; 2 Academy of Trauma Surgery (AUC—Akademie der Unfallchirurgie GmbH), 80538 Munich, Germany

**Keywords:** anti-osteoporotic medication, hip fractures, geriatric fractures

## Abstract

**Background**: Each year, hundreds of thousands of geriatric patients suffer proximal femur fractures, which often result in irreversible functional decline. Although anti-osteoporotic medications have been shown to reduce the incidence of fragility fractures, the effects of pharmacotherapy on fracture injury patterns, postoperative ambulatory capacity, and survival outcomes are not yet fully understood. **Methods**: We conducted a retrospective cohort study using the AltersTraumaRegister DGU ^®^ (ATR-DGU), a multicenter prospective registry spanning 196 certified geriatric trauma centers in Germany, Austria, and Switzerland (2016 through 2024). We included patients aged 70 years or older with isolated, surgically treated proximal femur fractures and stratified them by documented use of osteoporosis medication before the index fracture (OM group) versus no pre-fracture therapy (no-OM group). Primary endpoints were mortality during the acute hospital stay and at 120-day follow-up, as well as walking ability—defined as any functional ambulation versus none—at 7 and 120 days postoperatively. The main secondary endpoint was fracture morphology. We fitted multivariable logistic and linear regression models adjusted for age, sex, ASA classification, and anticoagulation; walking-ability models were additionally adjusted for pre-fracture ambulatory status. **Results**: Of 70,386 patients (mean age, 84.4 years; 70.4% female), 15,771 (22.4%) were receiving osteoporosis medication prior to the index fracture. Patients in the OM group were frailer with worse ASA Scores (80.3% vs. 72.5% with an ASA ≥3) and lower ambulation (62.5% vs. 50.4% requiring at least two crutches or not able to ambulate out of the home environment). Patients in the OM group had a 19% higher chance of sustaining a trochanteric versus a femoral neck fracture than patients in the no-OM group (51.9% vs. 46.8%; adjusted odds ratio 1.19; 95% CI, 1.15 to 1.24; *p* < 0.001). Pre-existing therapy showed no association with mortality during the acute hospital stay (adjusted OR, 0.93; 95% CI, 0.86 to 1.01; *p* = 0.098) or with walking ability at 7 days (adjusted OR, 1.03; 95% CI, 0.98 to 1.09; *p* = 0.198). At the 120-day follow-up (n = 19,544 in the adjusted model follow-up cohort), slightly higher odds of death were observed in the frailer OM group (adjusted OR, 1.12; 95% CI, 1.01 to 1.25; *p* = 0.04), though the lower confidence bound approached unity. A trend toward improved ambulation was seen in the OM group at 120-day follow-up (adjusted OR, 1.11; 95% CI, 0.99 to 1.25; *p* = 0.074). **Conclusions**: Pre-existing osteoporosis therapy was associated with lower odds of femoral neck versus trochanteric fracture patterns. A trend toward improved ambulation accompanied a slightly increased risk of mortality in patients with pre-existing osteoporosis therapy at 120-day follow-up, although this must be weighed against substantial loss to follow-up and residual confounding. More research is needed to understand the effects of anti-osteoporosis medications on patients who do suffer hip fractures despite osteoporotic therapy.

## 1. Introduction

A proximal femur fracture in geriatric patients is more than a musculoskeletal injury. It is, for many, the inflection point between independent living and institutional dependence, between ambulation and immobility, between survival and death within a year. One-year mortality after hip fracture ranges from 20 to 30 percent, and a substantial proportion of survivors never regain their pre-injury walking ability [1,2,3,4,5].

Anti-osteoporotic agents, including bisphosphonates, denosumab, teriparatide, and raloxifene, reduce incident fragility fractures in randomized trials [6,7,8]. A question that has not been addressed at scale in multicenter registry cohorts is whether, once a fracture occurs in a patient already receiving therapy, the medication modifies the nature of the injury and the clinical course itself. The proximal femur fails along predictable anatomical lines—through the femoral neck (intracapsular) or through the trochanteric region (extracapsular)—and these two fracture morphologies diverge in their surgical demands, complication profiles, and rehabilitation trajectories [9]. Whether pharmacologic modification of bone turnover shifts the fracture line from one pattern to the other has not been examined at scale [2,6].

Equally unresolved is whether pre-existing osteoporosis therapy alters the two outcomes that matter most to patients and their families: the ability to walk and the likelihood of surviving the months after surgery. Prior studies examining this question have been limited by sample size, single-center scope, or absence of standardized functional follow-up. What has been lacking is a cohort large enough and with structured longitudinal assessment, to detect (or exclude) clinically meaningful differences with adequate precision [9,10].

The ATR-DGU provides such a cohort [11]. Established in 2016 by the German Society for Trauma Surgery, the ATR-DGU prospectively captures demographic, surgical, and functional data—including standardized EQ-5D assessments—from elderly hip-fracture patients across nearly 200 certified geriatric trauma centers in Germany, Austria, Switzerland, and Luxembourg [11,12]. We used this registry to examine whether pre-existing osteoporosis therapy was associated with fracture type, postoperative ambulation, mortality, or health-related quality of life in geriatric patients with isolated proximal femur fractures [9].

## 2. Methods

### 2.1. Study Design and Data Source

We conducted a retrospective cohort study using prospectively collected data from the ATR-DGU. At the time of data extraction (May 2025), 197 certified geriatric trauma centers in Germany, Austria, Luxembourg, and Switzerland had contributed data. The registry records information at six defined time points: admission, the preoperative period, surgery, the seventh postoperative day, discharge or transfer, and a 120-day postoperative follow-up. The internal project code from the registry is ATR 2025-003.

### 2.2. Study Population

We screened all patients from 2016 to 2024 in the ATR-DGU registry. Eligible patients were aged 70 years or older and had sustained an isolated proximal femur fracture requiring surgery. We excluded patients with peri-prosthetic, peri-implant, or pathological fractures, those with concomitant injuries requiring treatment, and those with missing documentation of pre-fracture osteoporosis medication status. The remaining cohort was stratified into two groups: the OM group (documented pre-existing osteoporosis medication) and the no-OM group (no documented pre-fracture osteoporosis medication).

### 2.3. Exposure and Outcome Definitions

The primary exposure was the documented use of any osteoporosis medication before the index fracture. We assessed mortality at two registry-defined time points: death during the acute hospital stay (from admission to discharge from acute care) and death during the follow-up period extending up to 120 days postoperatively. The acute-stay mortality endpoint captures deaths at any point during the index hospitalization—which may extend well beyond 7 days—and should not be equated with a fixed 7-day mortality rate.

Walking ability was assessed at 7 days and 120 days postoperatively and dichotomized as any functional ambulation (independent or with aids) versus no functional walking ability. Secondary endpoints included fracture type distribution (femoral neck, trochanteric, subtrochanteric), health-related quality of life measured by the EQ-5D-5L index, length of acute hospital stay, and discharge destination [13]. For patients with documented EQ-5D-3L responses, we derived the EQ-5D-5L index through crosswalk mapping.

### 2.4. Statistical Analysis

We described continuous variables with means and standard deviations or medians and interquartile ranges, and categorical variables with absolute and relative frequencies. Following recommendations for large registry studies, we did not report *p*-values for baseline comparisons; in cohorts of this size, trivial differences routinely reach statistical significance, and distributional measures are more informative.

We assessed the association between pre-existing osteoporosis medication and fracture type using multinomial logistic regression, with femoral neck fracture as the reference category. For binary outcomes (mortality, walking ability, discharge destination), we used binomial logistic regression; for continuous outcomes (EQ-5D-5L index, length of stay), linear regression was used. All multivariable models were adjusted for age, sex, ASA classification (I–II, III, IV–V), and anticoagulation at admission. Walking-ability models carried an additional adjustment for pre-fracture ambulatory status, which reduced the available sample by 4549 patients who lacked baseline walking data. Discharge-destination models were additionally adjusted for pre-fracture living situation. We treated fracture type as a potential mediator and did not include it as a covariate in outcome models.

We also fitted unadjusted models for comparison. We report odds ratios or regression coefficients (β) with 95% confidence intervals and Wald-test *p*-values. A two-sided α of 0.05 was applied, but given the power of 70,000 observations, we prioritized effect magnitude and precision over dichotomous significance thresholds. We performed a prespecified subgroup analysis of trochanteric fractures by AO grade (A1, A2, A3). All analyses were conducted in R (Vienna, Austria, Version 4.5.0).

### 2.5. Missing Data

Missingness was low for most baseline variables: age (1041 of 70,386; 1.5%), sex (82; 0.1%), ASA classification (509; 0.7%), anticoagulation (1196; 1.7%), and fracture type (145; 0.2%). Pre-fracture walking ability was missing in 4549 patients (6.5%). The critical limitation was follow-up: 120-day data were available for only 23,049 of 70,386 patients (32.7%), drawn from 125 of 196 participating centers. This 67% attrition, concentrated at the institutional level, introduces selection bias that constrains the generalizability of all 120-day results. We performed no imputation; all models used complete cases for the respective variables.

### 2.6. Ethical Oversight and Data Governance

Data were collected in pseudo-anonymized forms under Good Clinical Practice guidelines and national data protection regulations. The scientific committee of the ATR-DGU approved the study protocol. Informed consent was obtained from all patients or their legal representatives at registry enrolment. No prospective protocol registration was performed for this retrospective analysis. The ATR-DGU data are owned by the AUC—Akademie der Unfallchirurgie GmbH; data-access requests may be submitted to the ATR-DGU scientific committee. The study was conducted in accordance with the principles outlined in the Declaration of Helsinki. For this study, only pooled, fully anonymized data were utilized, and approval was deemed unnecessary by the regional ethics committee.

## 3. Results

### 3.1. Study Population and Baseline Characteristics

The ATR-DGU contained 98,949 closed cases from 197 centers. After sequential exclusion of peri-prosthetic, peri-implant, and pathological fractures (leaving 82,507), patients with concomitant injuries (leaving 74,904), and those with missing osteoporosis-medication data (leaving 70,386 from 196 centers), we arrived at the primary analysis cohort. Of these patients, 15,771 (22.4%) had documented pre-existing osteoporosis medication and 54,615 (77.6%) did not. Follow-up data were available for 23,049 patients (32.7%) from 125 centers, of whom 5260 (22.8%) belonged to the OM group.

Table 1 summarizes the baseline characteristics. The two groups were similar in age (OM: 84.8 ± 6.3 years; no-OM: 84.2 ± 6.6 years) but differed in sex distribution, comorbidity burden, and functional status. Women comprised 78.2% of the OM group versus 68.1% of the no-OM group. An ASA classification of III or higher was present in 80.3% of the OM group versus 72.5% of the no-OM group. Before the fracture, only 27.6% of OM patients walked independently without aids, compared with 39.2% of no-OM patients; conversely, 41.4% of the OM group already required a walker, versus 32.5% of the no-OM group. One quarter of OM patients (25.7%) resided in a nursing home, compared with 19.1% of no-OM patients. Anticoagulation was more prevalent in the OM group (60.0% vs. 51.5%).

### 3.2. Fracture Morphology

Among 70,241 patients with a documented fracture type, the overall distribution was nearly evenly split between femoral neck (48.2%) and trochanteric (48.0%) fractures, with subtrochanteric fractures accounting for 3.9%. Hip fracture morphology differed in the OM group, where trochanteric fractures predominated (51.9% vs. 44.1% femoral neck). In the no-OM group, femoral neck fractures predominated (49.3% vs. 46.8% trochanteric).

Unadjusted multinomial regression (n = 70,241) confirmed this pattern: the odds of sustaining a trochanteric rather than a femoral neck fracture were 24% higher in patients receiving osteoporosis therapy (OR, 1.24; 95% CI, 1.20 to 1.29; *p* < 0.001); the odds of a subtrochanteric fracture were 18% higher (OR, 1.18; 95% CI, 1.07 to 1.29; *p* < 0.001). After adjustment for age, sex, ASA classification, and anticoagulation (n = 67,488), the association attenuated modestly but remained clear: adjusted OR for trochanteric versus femoral neck fracture, 1.19 (95% CI, 1.15 to 1.24; *p* < 0.001); for subtrochanteric versus femoral neck fracture, 1.13 (95% CI, 1.03 to 1.24; *p* = 0.012). All major endpoints are summarized in Table 2.

### 3.3. Mortality

Pre-existing osteoporosis therapy showed no association with death during the acute hospital stay. Unadjusted OR was 0.98 (95% CI, 0.91 to 1.07; *p* = 0.676; n = 67,604). After multivariable adjustment (n = 65,160), the point estimate shifted slightly below unity, but the confidence interval spanned the null (adjusted OR, 0.93; 95% CI, 0.86 to 1.01; *p* = 0.098).

At 120 days, a different pattern emerged. Unadjusted, the OM group had 16% higher odds of death (OR, 1.16; 95% CI, 1.05 to 1.28; *p* = 0.004; n = 20,307). Adjustment narrowed the estimate and just preserved nominal significance (OR, 1.12; 95% CI, 1.01 to 1.25; *p* = 0.04; n = 19,544). The lower confidence bound of 1.01 borders on unity, and the analysis was conducted in a subgroup comprising only 28% of the original cohort.

### 3.4. Walking Ability

At 7 days after surgery, patients in the OM group appeared less likely to walk than those in the no-OM group in the unadjusted analysis (OR, 0.88; 95% CI, 0.84 to 0.92; *p* < 0.001; n = 67,092). This apparent disadvantage vanished upon adjustment for age, sex, ASA, anticoagulation, and—critically—pre-fracture walking status (adjusted OR, 1.03; 95% CI, 0.98 to 1.09; *p* = 0.198; n = 60,781). The reduced sample reflects the exclusion of 4549 patients lacking baseline walking data.

At 120 days, the same confounding pattern held. The unadjusted OR of 0.90 (95% CI, 0.81 to 0.99; *p* = 0.035; n = 18,419) reversed direction after adjustment to 1.11 (95% CI, 0.99 to 1.25; *p* = 0.074; n = 17,123)—trending towards a modest benefit.

### 3.5. Health-Related Quality of Life

At 7 days, the adjusted EQ-5D-5L index was marginally lower in the OM group (β, −0.02; 95% CI, −0.03 to −0.02; *p* < 0.001; n = 35,809). By 120 days, the direction reversed: the OM group scored 0.03 points higher (β, 0.03; 95% CI, 0.01 to 0.05; *p* = 0.007; n = 9572). On a scale spanning 1.661 units (−0.661 to 1.000), this difference amounts to 1.8% of the total range.

### 3.6. Length of Stay and Discharge Destination

The adjusted difference in length of acute hospital stay between groups was negligible (β, 0.06 days; 95% CI, −0.10 to 0.22; *p* = 0.433; n = 67,163). Discharge to home or assisted living was less likely in the OM group after adjustment for pre-fracture living situation (adjusted OR, 0.91; 95% CI, 0.87 to 0.95; *p* < 0.001; n = 61,068). By 120 days, this disparity had resolved: among survivors, the adjusted OR for home residence was 0.99 (95% CI, 0.89 to 1.10; *p* = 0.84; n = 16,956).

All models were adjusted for age, sex, ASA classification (I–II, III, IV–V), and anticoagulation at admission. Walking-ability models additionally adjusted for pre-fracture walking ability. Discharge models additionally adjusted for pre-fracture living situation. Reference group: no osteoporosis medication. OR denotes odds ratio; β denotes unstandardized regression coefficient. The 120-day residence model includes only patients alive at follow-up.

## 4. Discussion

In this registry-based study of 70,386 elderly patients with proximal femur fractures, an association between pre-existing osteoporosis therapy and a trochanteric fracture morphology was seen: patients receiving anti-osteoporotic agents before their fracture sustained proportionally more intertrochanteric and subtrochanteric fractures and fewer femoral neck fractures. This association persisted after adjustment and was robust across both the unadjusted and multivariable models, with a 19% and 13% increase in the adjusted odds of trochanteric and subtrochanteric fractures versus femoral neck fracture patterns, respectively. This difference in fracture morphology seen in the OM group is quite interesting and the decrease in odds of an intracapsular fracture in the OM group has to our knowledge not been described before.

The data show that femoral neck fractures are less likely and trochanteric fracture patterns are more likely in patients with osteoporosis treatment. While we cannot draw mechanistic conclusions with our study data, and we lack the knowledge as to which specific osteoporotic medications the OM group received and for how long, several studies have evaluated how bone mineral density (BMD) and biomechanical properties around the hip change with various anti-osteoporosis agents. For example, Winzenrieth et al. assessed spatial changes in 3D dual-energy X-ray absorptiometry (DXA) cortical endpoints in trabecular BMD, cortical thickness, and biomechanical properties versus placebo for 18 months [14]. In patients treated with teriparatide and especially abaloparatide, new bone formation as determined by increases in cortical surface bone mineral density, cortical volumetric BMD, and cortical thickness were more pronounced in the femoral neck. Further, biomechanical properties showed greater relative improvement in the femoral neck and lower shaft regions in patients treated with teriparatide and abaloparatide vs. placebo. Another study measuring BMD in patients treated with abaloparatide vs. placebo found similar results [15]. We postulate that some osteoporosis therapies may provide a relatively larger protective effect at the level of the femoral neck, thus providing more protection in the femoral neck region in low energy falls.

The implications of such a shift in fracture morphology may be significant, as the literature has shown that lower incidence of femoral neck fractures versus trochanteric fractures has a positive impact on length of stay, hospital complications, mobilization and QoL [16,17,18,19]. In our study population, however, the observed fracture redistribution did not translate into significant short-term differences in mortality or walking ability, correlating with a previous study from Mangram et al. [20]. However, we did see a trend toward lower initial mortality with an adjusted OR for acute-stay mortality of 0.93 (95% CI, 0.86 to 1.01, *p* = 0.098) in the OM group. We speculate that this trend may be explained by the decreased risk in femoral neck fractures, as morbidity and mortality have been shown to be lower in patients who can be treated with intramedullary nails versus hip arthroplasty [19,21]. We highlight, however, that our data showed no statistically significant effect, and cannot provide a mechanistic explanation for these findings. If in the future, it can be proven that the use of osteoporosis medication decreases the risk of femoral neck fractures, it would have significant, likely positive implications for the management of geriatric hip fractures.

The 120-day mortality data require scrutiny. The adjusted OR of 1.12 (95% CI, 1.01 to 1.25; *p* = 0.04) reached nominal significance, but several factors urge restraint. The lower confidence bound barely crosses unity. The analysis rests on 19,544 patients—only 28% of the baseline cohort and a subset of the 23,049 patients (32.7%) with any 120-day follow-up, further reduced by covariate missingness. Perhaps more importantly, the OM group was much frailer and suffered a higher comorbidity burden as seen by the confounders we measured—while we adjusted for these confounders, they serve only as proxies for a latent frailty gradient, which is unlikely to be fully accounted for in our multi-variate analysis. As the literature has exhaustively shown, frailty and lower pre-fracture mobility strongly correlate with worsened outcomes in geriatric hip-fracture patients [17,18,22,23].

As mentioned above, immediate postoperative ambulation was similar in both groups, even after adjusting for pre-fracture walking ability as a covariate, as the point estimates shifted above 1.00 to 1.03 and 1.11, respectively. However, at 120 days, a strong trend toward improved ambulation was seen in the OM group (adjusted OR, 1.11; 95% CI, 0.99 to 1.25; *p* = 0.074). This occurred despite much worse pre-fracture mobility in the OM group. The data does not elucidate why this may be and there are many potential factors on which we may speculate. One of many possibilities may be the decreased risk of re-fracture or subsequent fracture for patients under osteoporosis treatment [1,6,10]. This is, however, a complex topic, as different anti-osteoporosis agents have a plethora of effects, including cardiovascular health, mortality, and sarcopenia. To comment further would overstep the scope of the current study.

Finally, with respect to quality of life, we did see a small benefit in patients under osteoporosis treatment based on EQ-5D-5L self-reported outcomes (β, 0.03; 95% CI, 0.01 to 0.05; *p* = 0.007). This result was statistically significant but is clinically irrelevant as the EQ-5D-5L spans 1.661 index units. A difference of 0.03 thus represents only 1.8% of the full range. Published thresholds for minimal clinically important differences in the EQ-5D-5L in musculoskeletal populations have been estimated at 0.04 to 0.08 [24].

This study has several limitations. First, as already touched upon above, the observational design precludes causal inference. As stated above, unmeasured confounders may bias the associations. One important example is confounding by indication, as patients with osteoporosis medication are likely to be inherently different to those without. This may partially explain why the OM group was the frailer of the study groups. Second, the registry does not distinguish between anti-osteoporosis agents, nor do we have data on length of, or adherence to treatment. This therefore prevents evaluation of drug-specific effects. Third, the acute-stay mortality endpoint captures all in-hospital deaths regardless of hospitalization duration and is not a fixed 7-day rate. Fourth, the 32.7% follow-up rate at 120 days limits the external validity of all medium-term findings and introduces selection bias. Further, the 120-day follow-up data were drawn from only 125 of 196 centers; the remaining 71 centers contributed no follow-up data. The 67% attrition was concentrated at the institutional level, meaning that entire hospitals contributed no follow-up data. Whether the patients available at 120-day follow-up resemble those lost to follow-up is unknown; the resulting selection bias could inflate, attenuate, or reverse the true association. Unfortunately, data on any potential differences in patients from centers reporting or not reporting 120-day follow-up are not available. Further, a sensitivity analysis with respect to institutions that collected 120-day follow-up was not performed. Fifth, the large sample size generates statistical power to detect effects of no clinical consequence; we have emphasized effect sizes and confidence intervals over dichotomous significance throughout. Sixth, the EQ-5D-5L captures composite health-related quality of life and may lack sensitivity for fracture-specific functional deficits. Seventh, absolute event counts for mortality and walking-ability endpoints were not available from the registry data extraction; readers should note that event rates per group cannot be derived from the odds ratios alone.

## 5. Conclusions

In conclusion, among over 70,000 elderly patients with proximal femur fractures, patients under pre-existing osteoporosis therapy had lower odds of a femoral neck fracture versus a trochanteric fracture. This was accompanied by a trend toward improved ambulation at 120-day follow-up. A slightly increased risk of mortality in patients with pre-existing osteoporosis therapy at 120-day follow-up must be weighed against substantial loss to follow-up and residual confounding. While these findings only potentially suggest that osteoporosis treatment may also be of benefit to patients who do suffer a hip fracture despite osteoporosis therapy, we find that the potential implications of these findings may be significant and urge that more research is warranted in this important field.

## Figures and Tables

**Table 1:** Baseline characteristics of patients with proximal femur fractures, according to pre-existing osteoporosis medication.

Characteristic	Total (N = 70,386)	No OM (n = 54,615)	OM (n = 15,771)
Age at admission—yr			
Mean ± SD	84.35 ± 6.55	84.20 ± 6.62	84.83 ± 6.28
Median (IQR)	85 (80–89)	84 (80–89)	85 (81–89)
Female sex—no./total no. (%)	49,460/70,304 (70.4)	37,146/54,552 (68.1)	12,314/15,752 (78.2)
ASA classification—no./total no. (%)			
I–II	18,000/69,877 (25.8)	14,905/54,204 (27.5)	3095/15,673 (19.8)
III	47,143/69,877 (67.5)	35,812/54,204 (66.1)	11,331/15,673 (72.3)
IV–V	4734/69,877 (6.8)	3487/54,204 (6.4)	1247/15,673 (8.0)
Walking ability before fracture—no./total no. (%)			
Independent without aids	24,091/65,837 (36.6)	20,018/51,101 (39.2)	4073/14,736 (27.6)
Walking stick or crutch	6773/65,837 (10.3)	5323/51,101 (10.4)	1450/14,736 (9.8)
Two crutches or walker	22,685/65,837 (34.5)	16,586/51,101 (32.5)	6099/14,736 (41.4)
Limited ability in apartment	10,370/65,837 (15.8)	7757/51,101 (15.2)	2613/14,736 (17.7)
No functional walking ability	1918/65,837 (2.9)	1417/51,101 (2.8)	501/14,736 (3.4)
Living situation—no./total no. (%)			
Home	54,383/69,495 (78.3)	43,016/53,906 (79.8)	11,367/15,589 (72.9)
Nursing home	14,300/69,495 (20.6)	10,299/53,906 (19.1)	4001/15,589 (25.7)
Hospital or other	812/69,495 (1.2)	591/53,906 (1.1)	221/15,589 (1.4)
Anticoagulation at admission—no./total no. (%)	36,951/69,190 (53.4)	27,672/53,712 (51.5)	9279/15,478 (60.0)
Fracture type—no./total no. (%)			
Femoral neck (medial/intracapsular)	33,828/70,241 (48.2)	26,888/54,495 (49.3)	6940/15,746 (44.1)
Trochanteric	33,692/70,241 (48.0)	25,519/54,495 (46.8)	8173/15,746 (51.9)
Subtrochanteric	2721/70,241 (3.9)	2088/54,495 (3.8)	633/15,746 (4.0)

OM denotes osteoporosis medication before fracture. ASA denotes American Society of Anesthesiologists physical status classification. Values are no./total no. with valid data (%) or mean ± SD. Percentages are based on valid observations per variable. No *p*-values are reported for baseline comparisons.

**Table 2:** Adjusted associations between pre-existing osteoporosis medication and clinical outcomes.

Outcome	OR or β	95% CI	*p* Value
Fracture Type (Multinomial; Ref: Femoral Neck)			
Trochanteric vs. femoral neck	1.19	1.15 to 1.24	<0.001
Subtrochanteric vs. femoral neck	1.13	1.03 to 1.24	0.012
Mortality during acute hospital stay	0.93	0.86 to 1.01	0.098
Mortality during follow-up (≤120 days)	1.12	1.01 to 1.25	0.04
Walking ability, 7 days post-op	1.03	0.98 to 1.09	0.198
Walking ability at follow-up (≤120 days)	1.11	0.99 to 1.25	0.074
Discharge to home/assisted living	0.91	0.87 to 0.95	<0.001
Residence at home at 120 days	0.99	0.89 to 1.10	0.84
EQ-5D-5L index, 7 days post-op (β)	−0.02	−0.03 to −0.02	<0.001
EQ-5D-5L index, 120 days post-op (β)	0.03	0.01 to 0.05	0.007
Length of acute hospital stay, days (β)	0.06	−0.10 to 0.22	0.433

## Data Availability

The datasets presented in this article are not readily available because they are protected by the registry for data privacy reasons. Requests to access the datasets should be directed to Henrik Teuber.
